# NK cells negatively regulate CD8 T cells via natural cytotoxicity receptor (NCR) 1 during LCMV infection

**DOI:** 10.1371/journal.ppat.1007725

**Published:** 2019-04-17

**Authors:** Katharina Pallmer, Isabel Barnstorf, Nicolas S. Baumann, Mariana Borsa, Stipan Jonjic, Annette Oxenius

**Affiliations:** 1 Institute of Microbiology, ETH Zürich, Zürich, Switzerland; 2 Department of Histology and Embryology, Faculty of Medicine, Rijeka, Croatia; University of Massachusetts Medical School, UNITED STATES

## Abstract

Besides their function in recognizing cancerous and virally infected cells, natural killer (NK) cells have the potential to shape adaptive immune responses. However, the mechanisms employed by NK cells to negatively regulate virus-specific CD8 T cell responses remain to be fully defined. Using activating receptor natural cytotoxicity receptor (NCR) 1 deficient (NCR1^gfp/gfp^) mice, we found increased numbers of virus-specific CD8 T cells, leading to enhanced virus control during acute LCMV infection. Furthermore, virus-specific CD8 T cells were more activated in the absence of NCR1, resulting in exacerbated immunopathology, documented by weight loss, and superior virus control early during chronic LCMV infection. Transfer experiments of virus-specific CD8 T cells into NCR1 deficient hosts revealed a direct cross talk between NK and CD8 T cells. Studies on the splenic microarchitecture revealed pronounced disorganization of T cells in infected NCR1^gfp/gfp^ mice, resulting in enhanced immunopathology and disruption of the T cell niche upon chronic LCMV infection. Our data show a novel pathway employed by NK cells to regulate antiviral CD8 T cell responses, namely direct recognition and elimination of activated CD8 T cells via NCR1 early during infection to protect the host from an overshooting T cell response.

## Author summary

LCMV, which is part of the *Arenaviridae family*, is a well-established mouse model for acute and chronic virus infections, and it has allowed the identification of many immunological principles that were subsequently confirmed in human infections, such as CTL escape or CD8 T cell exhaustion. NK cells belong to the first line defense, being activated early following infection or exposure to malignant cells, and mediate their antiviral or anti-tumoral effect by direct cytotoxicity and inflammatory cytokine secretion. While NK cells are dispensable for control of LCMV, NK cells have the potential to shape adaptive immunity by regulating T cell responses. The absence of NK cells leads to increased T cell immunity and thereby, to faster eradication of the virus. However, the detailed mechanisms of how NK cells control antiviral T cell responses is still poorly defined. Here, we identified the activating NK cell receptor NCR1 to be involved in the regulation of CD8 T cell responses during acute and chronic LCMV infection. The absence of NCR1 led to a more robust CD4 and CD8 T cell response and to superior viral control in acute and chronic LCMV infections. However, the increased CD8 T cell responses led to severe immunopathology in the setting of chronic infection. Hence, NK cells curtail CD8 T cell responses to protect the host from immunopathological damage in an NCR1 dependent manner.

## Introduction

Natural killer (NK) cells play an essential role in eliminating cancerous and virus infected cells, they co-express Eomesodermin (EOMES) and Tbx21; T-box expressed in T cells (T-bet) and are able to produce cytotoxic mediators such as perforin/ granzyme B and Interferon (IFN)γ [1, 2]. Compared to T and B cells, NK cells stochastically express various germ-line encoded activating and inhibitory receptors. The net balance of stimuli perceived from activating and inhibitory receptors decides whether an NK cell is activated and exerts its killing function, or is inhibited. NK cell mediated lysis of healthy cells is tightly regulated by the constitutive expression of ligands for inhibitory receptors such as major histocompatibility complex (MHC) class I, binding to Ly49A, C, D in mice [3]. In contrary, activating ligands are actively up-regulated upon tumorigenesis, DNA damage or virus infections, rendering those cells susceptible for NK cell mediated lysis. Such activating ligands comprise stress induced surface proteins such as MHC class I polypeptide-related sequence (MIC) A, MICB, members of the UL16-binding protein (ULBP) protein family and murine ULBP-like transcript 1 (MULT-1), binding to the activating receptor NKG2D [3]. NK cells are also involved in the recognition of infected cells by sensing viral proteins via their activating receptors. The engagement of the murine Cytomegalovirus (MCMV) encoded protein m157 with the activating receptor Ly49H leads to NK cell activation and eradication of the infected cell [4]. Moreover, viral hemagglutinin (HA) and neuraminidase (NA) derived from influenza virus, poxvirus, sendai virus and new-castle disease virus are recognized by the activating receptor natural cytotoxicity receptor (NCR) 1, which is NKp46 in humans, leading to effective lysis of the virally infected cells [5–8]. Previously, it was reported that NCR1 is also essential for the recognition of fungal derived ligands from *Candida glabrata*, leading to systemic clearance of the fungus [9]. In a murine model for melanoma, the expression of NCR1 was crucial in order to control growth of metastases, indicating that NCR1 is involved in NK cell control of metastasis formation [10].

Besides recognizing and eliminating altered-self and virally infected cells, there is a growing body of evidence that NK cells can influence adaptive immune responses, resulting in either positive or negative effects on the emerging T cell response [11]. NK cells can act in an indirect manner via the modulation of antigen presenting cells (APCs) such as dendritic cells (DCs), which are able to potently activate naïve T cells. Bidirectional DC-NK cell crosstalk implies beneficial or detrimental effects. For instance, DCs are essential for NK cell activation in a direct manner, through receptor interaction (NKp30), or in an indirect manner via the secretion of cytokines such as TNF [12–14]. NK cells favor the maturation of DCs, leading to up-regulation of co-stimulatory molecules and increased cytokine secretion [14, 15]. Although NK cells can induce DC maturation, it was also reported that NK cells directly kill DCs, which is associated with poorer antigen presentation [16, 17]. Upon MCMV infection, the DC-derived secretion of IL-10 inhibited DC-NK cell cross talk, leading to hampered CD4 T cell responses and sustained replication of the virus [18]. However, previous studies also showed that NK cells have the capacity to directly kill CD4 and CD8 T cells via cytokine secretion and cytotoxicity. Studies using persistent lymphocytic choriomeningitis virus (LCMV) infection revealed that NK cells kill virus-specific T cells in a perforin dependent manner and thereby promote prolonged viral persistence [19–21]. The depletion of NK cells resulted in enhanced antigen presentation, a more robust T cell effector response and enhanced memory formation in a vaccine model [22]. In addition, the absence of NK cells led to a better antibody response as consequence of increased follicular helper CD4 T cells that are needed for germinal center reactions during acute LCMV infection [23]. Similarly, in chronic LCMV infection, NK cells were reported to reduce immunity, partly by inhibition of T helper cell and antibody responses [24]. Conversely, by limiting T cell responses, NK cells prevented fatal pathology during chronic LCMV infection [19]. Thus, NK-T cell interaction has profound consequences on the emerging T cell response and eventually, on the course of infection.

Activated T cells evolved mechanisms to protect themselves against NK cell mediated killing. Sensing type I IFN rescues CD4 and CD8 T cells, as T cells lacking the receptor for type I IFN (IFNAR) were directly eliminated by NK cells [25, 26] via NCR1 [26]. Of note, the cellular ligand for NCR1 on T cells remains elusive. Despite the understanding that NK cell depletion during LCMV infection promotes more robust T cell responses, the exact underlying mechanisms remain to be fully identified. In this study, we sought to address whether NCR1 influences T cell responses during acute and chronic LCMV infection. Using NCR1 deficient (NCR1^gfp/gfp^) mice, we found that the absence of NCR1 led to elevated virus-specific CD8 T cell numbers and accelerated control of the infection. During chronic LCMV infection, the frequency of virus-specific CD8 T cells was increased in absence of NCR1 and NCR1-deficient mice exhibited more pronounced immunopathology compared to wild type (WT) hosts. Moreover, pronounced splenic disorganization in absence of NCR1 compromised the T cell niche, likely provoking the reduction of total CD8 T cell numbers in LCMV infected NCR1-deficient mice. Transfer experiments revealed a direct NK-T cell cross talk with elimination of activated CD8 T cells in an NCR1-dependent manner. Our study unravels a mechanism whereby NK cells directly eliminate virus-specific CD8 T cells via NCR1. The absence of this regulation results in an overshooting CD8 T cell response that leads to better virus control, but also to aggravated immunopathology.

## Results

### Absence of activating receptor NCR1 does not alter NK cell responses upon LCMV infection

**Fig 1:**
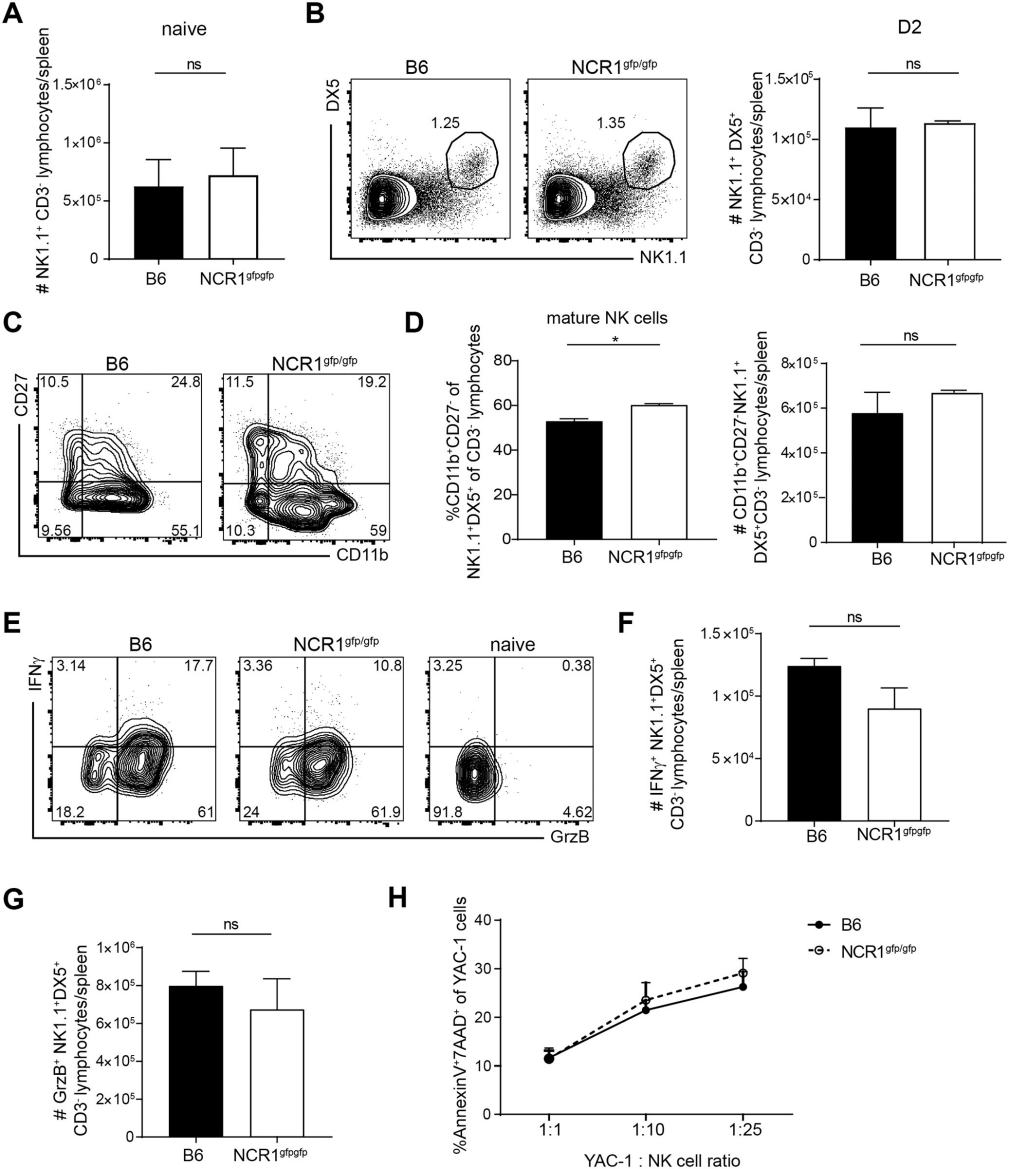
Absence of activating receptor NCR1 does not alter NK cell responses upon LCMV infection. (A) Total numbers of NK1.1^+^CD3^-^ lymphocytes in the spleen in naïve mice. Data shown are mean + SEM of n = 6–7 mice; pooled data from 2 experiments. ns, not significant (unpaired two-tailed *t*-test). (B-G) Analysis of splenic cells of C57BL/6 and NCR1^gfp/gfp^ mice 2 days p. LCMV WE infection. (B) Representative flow cytometry plots pre-gated on CD3^-^ and total number of CD3^-^ DX5^+^ NK1.1^+^ lymphocytes are shown. (C) Representative flow cytometry plots pre-gated on CD3^-^ DX5^+^ NK1.1^+^ and (D) frequency and total numbers of CD11b^+^ CD27^-^ are depicted. Data shown are mean + SEM of n = 3 mice representative of 2 independent experiments. ns, not significant; * p<0.05 (unpaired two-tailed *t*-test). (E-G) Splenocytes were incubated in presence of Brefeldin A for 5h at 37°C and IFNγ and Granzyme B (GrzB) expression was measured. (E) Representative flow cytometry plots pre-gated on CD3^-^ DX5^+^ NK1.1^+^ and total numbers of IFNγ (F) and Granzyme B (GrzB) producing cells (G). Data shown are mean + SEM of n = 3 mice representative of 2 independent experiments. ns, not significant (unpaired two-tailed t-test). H) NK cells from day 2 LCMV WE infected mice derived from WT and NCR1^gfp/gfp^ were MACS purified. 20.000 YAC-1 cells were co-cultured with purified NK cells at the indicated ratios of NK to target cells for 5h followed by cytometric analysis. The percentage of apoptotic Annexin V and 7-AAD double positive YAC-1 cells is shown. Data shown are mean + SEM of n = 3 mice representative of 2 independent experiments. ns, not significant (unpaired two-tailed *t*-test).

No alteration in development, frequency or effector functions are described for NK cells in NCR1^gfp/gfp^ mice at steady-state [27] compared to the NK cells of NKp46^icre^ mice, which were reported to be hyper-responsive [28]. To investigate whether NK cells lacking the activating receptor NCR1 are different from NCR1 sufficient NK cells during LCMV infection, we compared NK cells from C57BL/6 and NCR1^gfp/gfp^ mice. In naïve mice and in mice infected with LCMV WE for 2 days, NK cell numbers were comparable between C57BL/6 and NCR1^gfp/gfp^ mice (Fig 1A and 1B). NK cell maturation can be distinguished by different expression of surface markers in the following order of their development: CD11b^lo^ CD27^lo^, CD27^hi^ CD11b^lo^, CD27^hi^ CD11b^hi^ and CD27^lo^ CD11b^hi^ [29]. The maturation of murine NK cells in these four stages is accompanied by the acquisition of effector functions [30]. Even though the frequency of mature NK cells (CD27^lo^ CD11b^hi^) was slightly higher in absence of NCR1, the absolute number of mature NK cells was comparable 2 days after LCMV WE infection (Fig 1C and 1D). Total numbers of IFNγ and Granzyme B producing NK cells were similar in the absence or presence of NCR1 (Fig 1E–1G). YAC-1 cells, which are susceptible to NK cell mediated lysis, were co-cultured with NK cells derived from infected WT and NCR1^gfp/gfp^ mice. No differences of cytotoxicity between C57BL/6 and NCR1^gfp/gfp^ mice were observed (Fig 1H). Altogether, the quality and quantity of NK cells was comparable in NCR1^gfp/gfp^ and WT mice upon LCMV infection.

### Increase of virus-specific CD8 T cells in absence of NCR1 during acute LCMV infection

**Fig 2:**
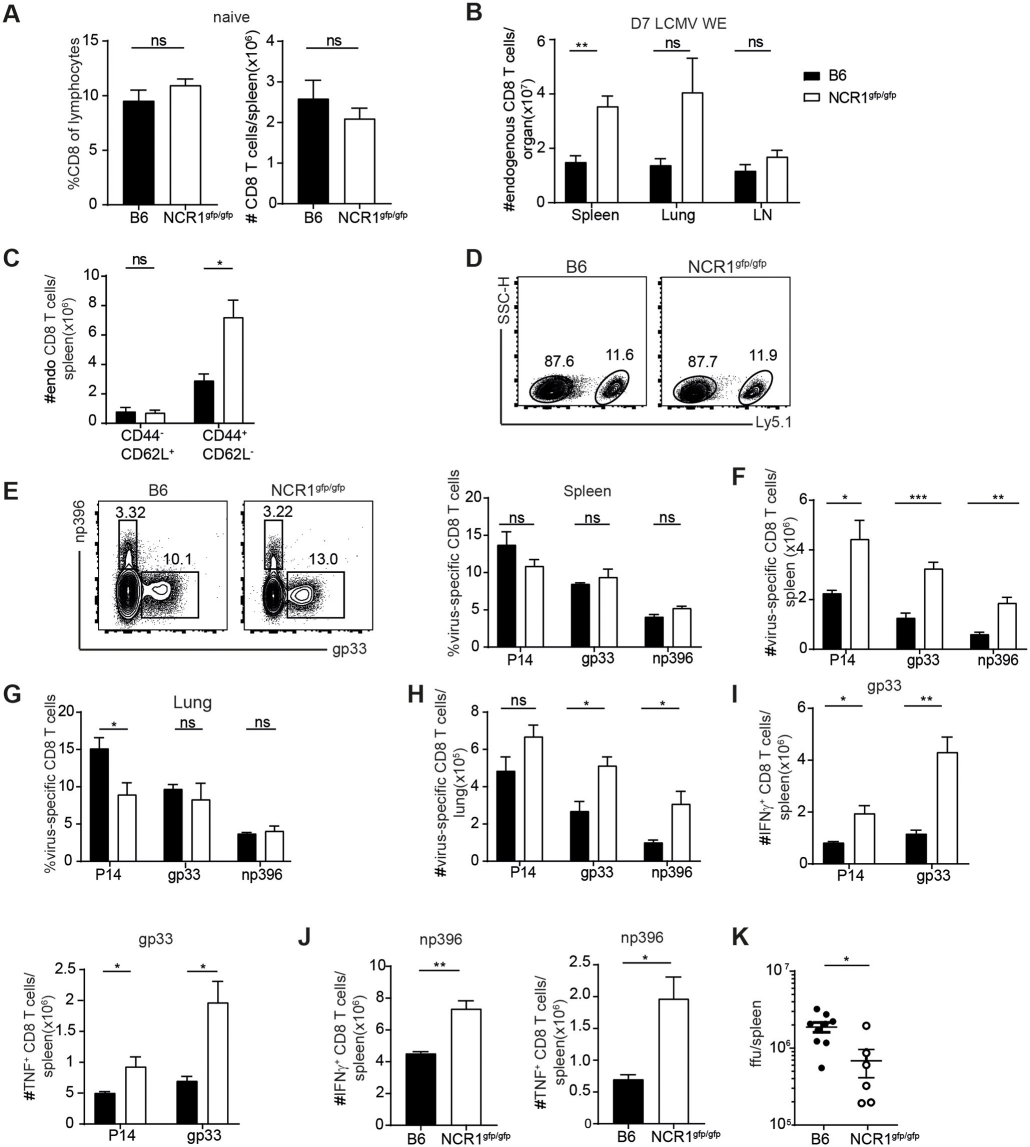
Increase of virus-specific CD8 T cells in absence of NCR1 during acute LCMV infection. (A) Frequency and total numbers of CD8 T cells in the spleen of naïve mice. Data shown are mean + SEM of n = 3 mice representative of 2 independent experiments. ns, not significant (unpaired two-tailed *t* test). (B) 1x10^3^ P14 T cells (Ly5.1^+^) were transferred into WT and NCR1^gfp/gfp^ mice followed by 200 ffu LCMV WE infection. After 7d, organs were harvested and flow cytometric analysis was performed. Number of CD8 T cells in indicated organs is shown. (C) 1x10^4^ P14 T cells (Ly5.1^+^) were transferred into WT and NCR1^gfp/gfp^ mice followed by 200 ffu LCMV docile infection. After 7d, organs were harvested and flow cytometric analysis was performed. Numbers of endogenous CD8 T cell subsets are shown. Data shown are mean + SEM of n = 5–6 mice pooled from 2 independent experiments. ns, not significant, * p<0.05 (unpaired two-tailed *t*-test). (D-J) 1x10^3^ P14 T cells (Ly5.1^+^) were transferred into WT and NCR1^gfp/gfp^ mice followed by 200 ffu LCMV WE infection. After 7d, organs were harvested and flow cytometric analysis was performed. (D) Representative flow cytometry plot pre-gated on CD8 T cells. (E) Representative flow cytometry plot pre-gated on endogenous CD8 T cells in the spleen. (F) Total number of transferred P14 (Ly5.1^+^), endogenous gp33-tet^+^ and endogenous np396-tet^+^ CD8 T cells in the spleen, (G) percentages and (H) total numbers in the lung. (I-J) Splenocytes were incubated in presence of Brefeldin A, gp33 and np396 peptide, respectively, for 6h at 37°C followed by intracellular cytokine staining. (I) Number of IFNγ and TNF producing CD8 T cells after gp33 stimulation is shown. (J) Number of IFNγ and TNF producing CD8 T cells after np396 stimulation is shown. Data shown are mean + SEM of n = 4 mice representative of 2 independent experiments. ns, not significant, * p<0.05,** p<0.01, *** p<0.001 (unpaired two-tailed t-test). (K) 1x10^3^ P14 T cells (Ly5.1^+^) were transferred into WT and NCR1^gfp/gfp^ mice followed by LCMV WE infection. After 5d, spleens were harvested and viral titers were determined. Data shown are mean ± SEM of n = 6–9 mice pooled from 2 independent experiments. ns, not significant, * p<0.05 (unpaired two-tailed *t*-test).

Previous studies have shown that NK cell depletion led to an increase of virus-specific CD8 T cells after LCMV infection [19, 21]. However, how exactly NK cells negatively regulate virus-specific T cells during LCMV infection remains unclear. Upon LCMV infection, activated virus-specific CD8 T cells lacking IFNAR, unable to sense type I IFN, are very efficiently killed by NK cells in an NCR1 dependent manner [26]. Here, we sought to address if virus-specific CD8 T cells, which are able to sense type I IFN, are also regulated by NK cells in an NCR1 dependent manner. One day prior to acute LCMV WE infection, we adoptively transferred gp33-specific transgenic CD8 T cells (P14 T cells, Ly5.1^+^) into C57BL/6 (Ly5.2^+^) and NCR1^gfp/gfp^ (Ly5.2^+^) mice and analyzed CD8 T cell responses in various organs 7 dpi. Numbers of CD8 T cells were comparable in naive NCR1^gfp/gfp^ and C57BL/6 mice (Fig 2A). Upon LCMV infection, total numbers of endogenous CD8 T cells were increased in absence of NCR1, both in NCR1^gfp/gfp^ mice and in C57BL/6 mice upon *in vivo* blocking of NCR1, using an αNCR1 antibody (ab) during infection (Fig 2B and S1A and S1B Fig). Only activated CD8 T cells (CD44^+^ CD62L^-^) were subjected to NCR1 mediated regulation by NK cells, because naïve CD8 T cells (CD44^-^ CD62L^+^) were comparable in NCR1^gfp/gfp^ and WT mice (Fig 2C). Collectively, these data suggest that activated CD8 T cells are negatively regulated by NK cells in an NCR1-dependent manner. Two recent publications demonstrated that absence of NCR1 leads to missing TNF-related apoptosis-inducing ligand (TRAIL) expression on the surface of NK cells [31, 32]. Therefore, we also tested TRAIL expression on NK cells of NCR1^gfp/gfp^ and αNCR1 treated C57BL/6 mice. Confirming the findings by Sheppard et al. and Turchinovich et al., TRAIL expression was absent on NK cells in absence of NCR1. However, blocking of NCR1 did not influence TRAIL expression (S2C and S2D Fig). As we had seen increased numbers of activated CD8 T cells in both NCR1-deficient and in NCR1-blocked WT mice, we concluded that TRAIL deficiency in NCR1^gfp/gfp^ mice was not responsible for enhanced T cell immunity.

We also quantified the frequency of transferred P14 T cells and of endogenous virus-specific CD8 T cells specific for the gp33 and np396 epitopes. Frequencies of both endogenous CD8 T cells and transferred P14 cells were comparable in presence or absence of NCR1 (Fig 2D and 2E and S1C–S1E Fig). However, total numbers of transferred P14 and endogenous gp33 and np396 specific CD8 T cells were doubled in spleen and lung in NCR1^gfp/gfp^ mice (Fig 2F and 2G) and increased in αNCR1 ab treated WT mice (S2D and S2E Fig). This numeric increase of LCMV-specific CD8 T cells is in line with the increased numbers of total activated CD8 T cells in the absence of NCR1 (Fig 2B and S1A and S1B Fig). Properdin, a modulator of the alternative pathway of the complement system, was described to be a ligand for NCR1 [33], but we could not detect increased presence of properdin on CD8 T cells in NCR1^gfp/gfp^ mice 4 days after LCMV WE infection (S3 Fig).

Consistent with the increased numbers of virus-specific CD8 T cells, total numbers of IFNγ and TNF producing CD8 T cells after gp33 and np396 stimulation were augmented in absence of NCR1 (Fig 2H–2J) and also increased upon NCR1 blockade in WT mice (S1F and S1G Fig). Elevated numbers of virus-specific CD8 T cells in NCR1^gfp/gfp^ mice led to enhanced virus control at day 5 p.i. (Fig 2K). Thus, NK cells dampened CD8 T cell responses during acute LCMV infection via NCR1, as ablation or blocking of NCR1 resulted in a more robust CD8 T cell response and superior control of the virus.

### Activated polyclonal CD8 T cells and virus-specific CD8 T cells are directly killed by NK cells via NCR1

To investigate if T cells are indirectly regulated by NK cells via DCs, we determined whether DCs were altered numerically or phenotypically in LCMV infected NCR1^gfp/gfp^ mice compared to WT controls. Specifically, we quantified expression levels of costimulatory molecules such as CD40, CD80 and CD86 on DCs 2 days after acute LCMV infection. No difference in DC number or expression of costimulatory molecules was observed (S4A–S4C Fig).

**Fig 3:**
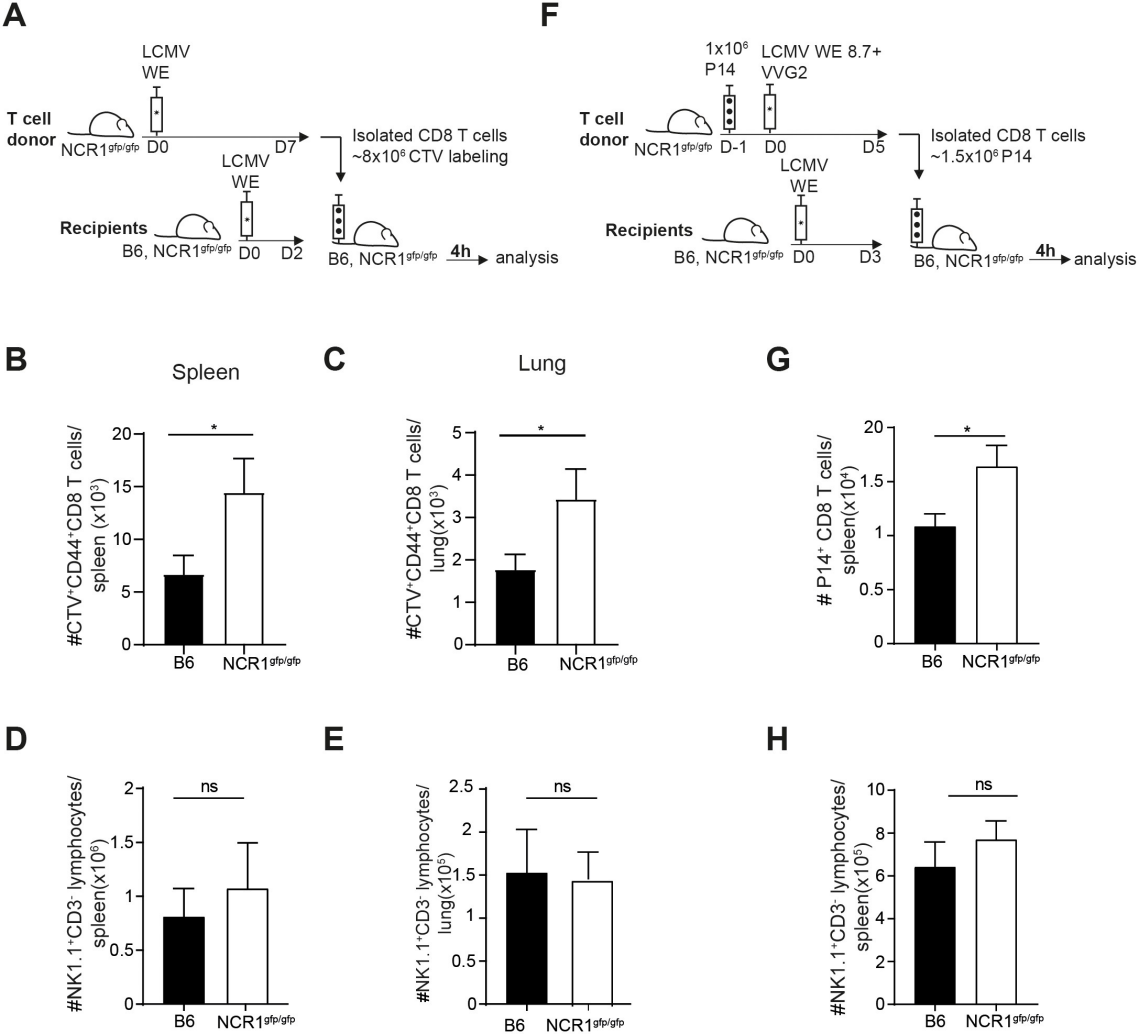
Activated polyclonal CD8 T cells and virus-specific CD8 T cells are directly killed by NK cells via NCR1. *(*A) Experimental setup of the *in vivo* killer assay used in B-E. NCR1^gfp/gfp^ were infected with LCMV WE and splenic CD8 T cells were positively MACS sorted and CTV labeled after 7d. Purified CD8 T cells were transferred into day 2 infected B6 and NCR1^gfp/gfp^ mice. After 4 h, spleen and lung tissue was harvested followed by flow cytometric analysis. Recovery of transferred CTV^+^ CD44^+^ CD8 T cells in the spleen (B) and in the lung (C). Data shown are mean + SEM of n = 12 mice pooled from 3 independent experiments. ns, not significant, * p<0.05 (unpaired two-tailed *t*-test). Total numbers of NK1.1^+^CD3^-^ cells in the spleen (D) and in the lung (E). Data shown are mean + SEM of n = 4 mice representative of 2 independent experiments. ns, not significant, (unpaired two-tailed t-test). (F) Experimental scheme used in G-H. 1x10^6^ P14 T cells (Ly5.1^+^) were transferred into NCR1^gfp/gfp^ mice followed LCMV WE8.7 and VVG2 co-infection. After 5d, splenic CD8 T cells were MACS sorted and transferred into day 3 infected B6 and NCR1^gfp/gfp^ mice. After 4 hours, spleens were harvested and P14 T cell (Ly5.1^+^) number was determined by flow cytometric analysis. (G) Recovery of P14 T cells (Ly5.1^+^) in the spleen. (H) Number of NK1.1^+^ CD3^-^ cells in the spleen are shown. Data shown are mean + SEM of n = 8 mice pooled from 2 independent experiments. ns, not significant, * p<0.05 (unpaired two-tailed *t*-test).

To address whether NK cells directly eliminated activated CD8 T cells in an NCR1-dependent manner, we performed *in vivo* cytotoxicity assays. For this, activated CD44^hi^ CD8 T cells were generated in NCR1^gfp/gfp^ mice by LCMV infection. On the peak of the T cell response, these target cells were isolated, labeled and transferred into infected recipients and target cell survival was quantified 4 hours later (Fig 3A). Indeed, target cell survival was higher in NCR1^gfp/gfp^ mice compared to WT recipients in spleen and lung (Fig 3B and 3C). NK cell numbers in spleen and lung were comparable in NCR1^gfp/gfp^ and WT recipients, indicating that the T:NK cell ratios were equal in WT and NCR1^gfp/gfp^ mice (Fig 3D and 3E). The same experiment was repeated with monoclonal LCMV-specific CD8 T cells as NK cell targets. To this end, naïve P14 T cells were transferred into NCR1^gfp/gfp^ recipient mice followed by antigen challenge using co-infection of LCMV WE 8.7 and recombinant vaccinia virus expressing LCMV GP (VVG2) that has been previously described [34]. P14 CD8 T cells were isolated 5 days after *in vivo* activation and were transferred into LCMV infected NCR1^gfp/gfp^ or WT hosts. Survival of transferred P14 T cells was assessed 4 hours later (Fig 3F). While NK cell numbers were comparable in NCR1^gfp/gfp^ and WT recipients, the survival of P14 T cells was significantly increased in NCR1^gfp/gfp^ recipients (Fig 3G and 3H). This data indicates that negative regulation of virus-specific CD8 T cells is mediated in a direct manner by NCR1-expressing NK cells.

### Highly activated and more functional CD8 T cells in absence of NCR1 during chronic infection

**Fig 4:**
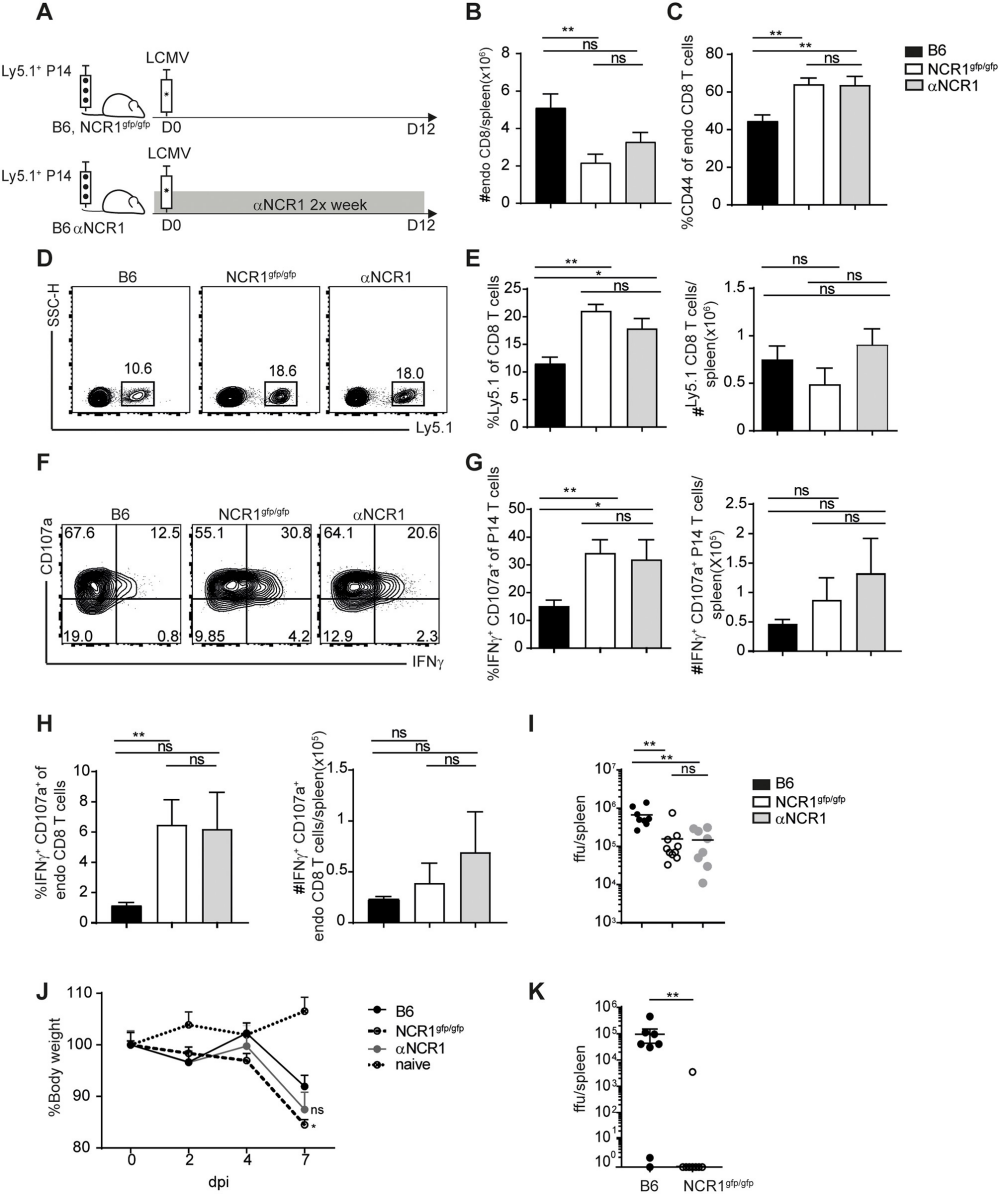
Highly activated and more functional CD8 T cells in absence of NCR1 during chronic infection. (A) Experimental setup used in B-I. 1x10^3^ P14 T cells (Ly5.1^+^) were transferred into WT, NCR1^gfp/gfp^ and αNCR1 blocked WT mice followed by LCMV docile infection. After 12d, organs were harvested and analyzed. (B) Total number of endogenous CD8 T cells and (C) frequency of CD44^+^ CD8 T cells pre-gated on endogenous CD8 T cells in the spleen. (D) Representative flow cytometry plot pre-gated on CD8 T cells. (E) Frequency and total numbers of P14 T cells (Ly5.1^+^) in the spleen. F-I) Splenocytes were incubated in presence of Brefeldin A, CD107a and gp33 peptide for 6h at 37°C followed by intracellular cytokine staining. (F) Representative flow cytometry plots pre-gated on P14 T cells (Ly5.1^+^). (G) Frequency and total numbers IFNγ^+^ CD107a^+^ P14 T cells (Ly5.1^+^). (H) Frequency and total numbers of IFNγ^+^ CD107a^+^ endogenous CD8 T cells in the spleen. I) Viral load in spleen. Data shown are mean ±SEM of n = 8–9 mice pooled from 3 independent experiments. ns, not significant, * p<0.05, ** p<0.01 (unpaired two-tailed t-test). (J) 1x10^3^ P14 T cells were transferred into WT, NCR1^gfp/gfp^ and αNCR1 blocked WT mice followed by LCMV docile infection. Weight curve normalized to initial body weight. Data shown are mean + SEM of n = 4–7 mice pooled from 2 independent experiments. ns, not significant, * p<0.05 (two way ANOVA followed by Sidak’s multiple comparison test). (K) B6 and NCR1^gfp/gfp^ mice were infected with 1.5x10^4^—2x10^4^ ffu LCMV docile. Spleens were harvested 12 dpi and viral load was determined. Data shown are mean of n = 8 pooled from 2 independent experiments. ns, not significant, * p<0.05, ** p<0.01 (unpaired Mann-Whitney U test).

NK cell depletion in a setting of a chronic infection may lead to viral clearance [19, 21]. Therefore, after investigating the role of NCR1 in acute virus infection, we examined how the absence of NCR1 influences CD8 T cell responses in chronic LCMV infection. After transfer of P14 T cells into C57BL/6, NCR1^gfp/gfp^ and C57BL/6 mice treated with αNCR1 ab, we quantified the CD8 T cell response after infection with LCMV docile 12 days post infection (Fig 4A). Surprisingly, the number of total endogenous CD8 T cells was lower in absence of NCR1, but CD8 T cells appeared to be highly activated (CD8^+^ CD44^+^) at the same time (Fig 4B and 4C). The frequencies of virus-specific CD8 T cells were highly increased in NCR1^gfp/gfp^ and in anti-NCR1-blocked C57BL/6 mice compared to WT mice (Fig 4D and 4E). In addition, LCMV-specific CD4 T cells (TCR transgenic CD4 T cells with specificity for the LCMV gp_61-80_ epitope, Smarta T cells) were increased in frequency and numbers upon chronic LCMV infection (S5 Fig). Due to decreased overall numbers of CD8 T cells, P14 T cell numbers were not increased in the absence of NCR1 (Fig 4E). The frequency of CD8 T cells being able to degranulate or to secrete IFNγ was enhanced within endogenous gp33-specific and transferred P14 T cells in NCR1^gfp/gfp^ and NCR1 blocked WT mice compared to untreated C57BL/6 mice (Fig 4F–4H). Heterozygous NCR1^gfp/+^ mice and isotype (αIgG1 κ) treated WT mice did not show an increased CD8 T cell response upon chronic LCMV infection (S6 Fig). Consistent with a more robust CD8 T cell response in absence of NCR1, viral titers were decreased in mice lacking NCR1 or receiving NCR1 blockade (Fig 4I) and NCR1 deficient mice showed a pronounced weight loss of about 15%, in comparison to WT mice that lost less of their initial weight (Fig 4J). When mice were infected with an intermediate dose of LCMV docile, we observed that most NCR1^gfp/gfp^ mice were able to completely control the infection, whereas B6 mice showed still high titers at 12 dpi in the spleen (Fig 4K). This demonstrates that the absence of NCR1 leads to faster viral control compared to NCR1 expressing WT mice, also without additional transfer of transgenic CD8 T cells upon LCMV docile infection. Taken together, NK cells have the capacity to control virus-specific T cell responses by killing activated virus-specific CD4 and CD8 T cells during chronic LCMV infection in an NCR1 dependent manner.

### Disrupted T cell organization in spleens of NCR1 deficient mice during LCMV infection

**Fig 5:**
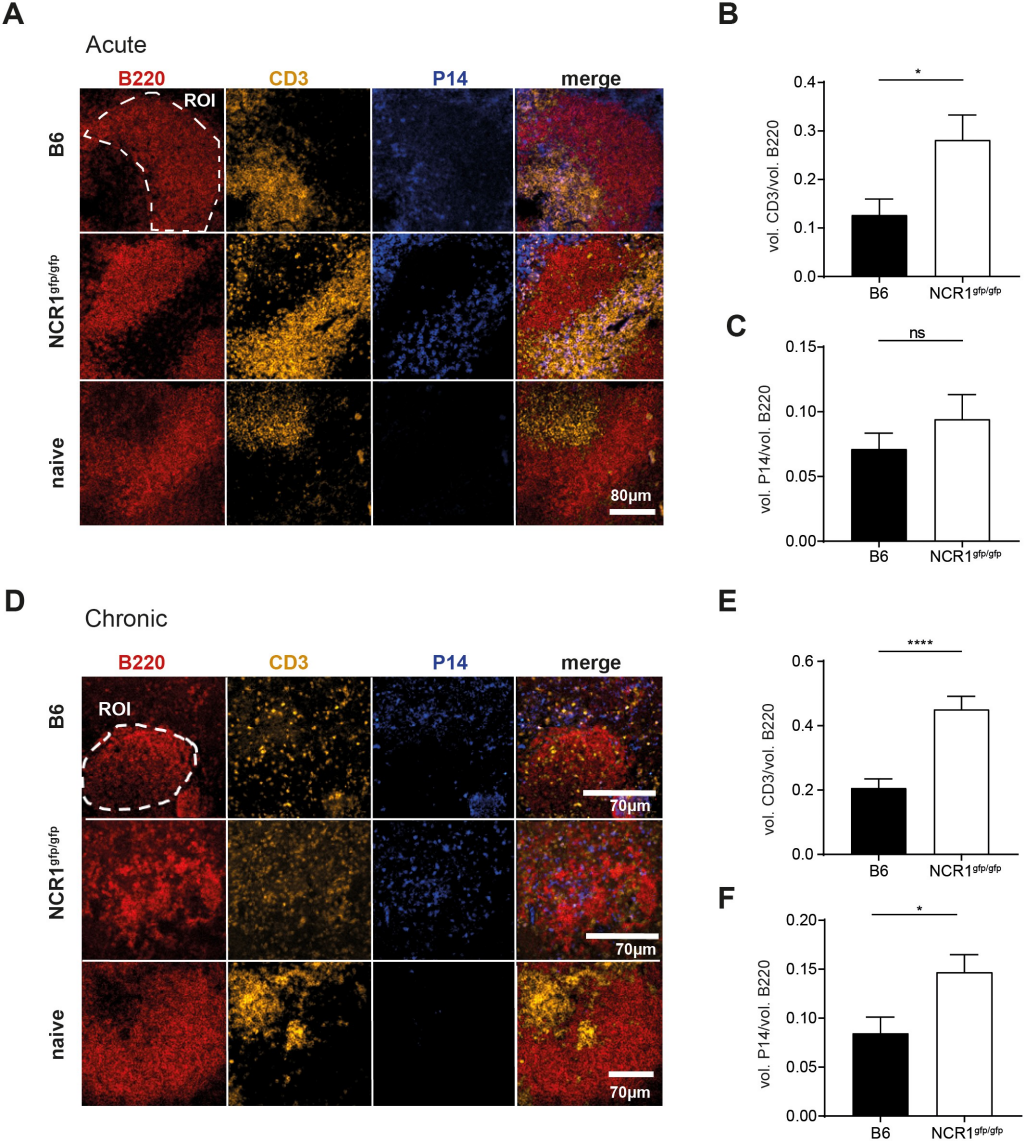
Severe disruption of splenic architecture in absence of NCR1 during chronic LCMV infection. (A-C) 2x10^4^ P14 T cells (Ly5.1^+^) were transferred into WT and NCR1^gfp/gfp^ mice followed by LCMV WE infection. After 8 days, spleens were harvested and prepared for confocal microscopy and pictures were taken with a 10x objective. (A) Overview of the splenic sections. Region of interest (ROI), dashed line. Scale bar 80 μm (B-C) Ratio of MFI of volume of B cell zones (B220^+^) and the volume of MFI of T cells found within B cell zone was determined. Shown is the ratio of volume between B cell zone-localized (B) CD3^+^ cells/ (C) P14 T cells (Ly5.1^+^) and the volume of the B cell zone, respectively. Data shown are mean + SEM of 61–66 B cell zones derived from 4–5 mice pooled from 2 experiments. ns, not significant, * p<0.05 (unpaired two-tailed *t* test). (D-F) 2x10^3^ P14 T cells (Ly5.1^+^) were transferred into WT and NCR1^gfp/gfp^ mice followed by LCMV docile infection. After 8 days, spleens were harvested and prepared for confocal microscopy. (D) Overview of the splenic sections. Region of interest (ROI), dashed line. Scale bar 70 μm (E-F) Ratio of MFI of volume of B cell zones (B220^+^) and the volume of MFI of T cells found within B cell zone was determined. Shown is the ratio of volume between B cell zone-localized (E) CD3^+^ cells/ (F) P14 T cells (Ly5.1^+^) and the volume of the B cell zone, respectively. Data shown mean ±SEM of 54–97 B cell zones from 8–9 mice pooled from 3 experiments * p<0.05, **** p<0.0001 (unpaired two-tailed *t*-test).

LCMV WE and docile are known to infect myeloid cells such as macrophages and dendritic cells, but also stromal cells including fibroblastic reticular cells, leading to cytotoxic T cell-mediated killing of infected cells. This results in destruction of the structure of secondary lymphoid organs, consisting of distinct B and T cell zones [35–37]. Naïve C57BL/6 and NCR1^gfp/gfp^ mice had the same amount of CD8 T cells at steady-state (Fig 2A). During the course of an acute LCMV WE infection, CD8 T cells expanded 2-fold more in NCR1^gfp/gfp^ compared to WT mice (Fig 2B), including virus-specific TNF and IFNγ-producing CD8 T cells. To evaluate whether the enhanced CD8 T cell response in absence of NCR1 affected the splenic architecture, we analyzed splenic tissue after 8 days of acute LCMV WE infection. In naïve mice, we observed a clear separation between B cell zone (B220) and T cell zone (CD3) (Fig 5A). Although some T cells were found to be dislocated to the B cell zone in LCMV-infected C57BL/6 mice, the number of B cell zone dislocated CD3 T cells was significantly increased in NCR1^gfp/gfp^ mice (Fig 5B). However, the number of P14 T cells located within the B cell zone was comparable in NCR1^gfp/gfp^ and WT mice (Fig 5C). Thus, the splenic architecture was largely maintained during acute infection with a small increase in splenic microarchitecture disruption in NCR1^gfp/gfp^ mice.

Next, we investigated the lymphoid structure in spleens of C57BL/6 and NCR1^gfp/gfp^ mice following a chronic LCMV infection. Here, the T cell zone could not be demarcated anymore (Fig 5D) and the B cell zones were highly populated by T cells (Fig 5E), and also by virus-specific P14 T cells in absence of NCR1 (Fig 5F). This effect held also true for WT mice, but to a significantly reduced extent. This observation might explain the overall reduction in CD8 T cell numbers in NCR1^gfp/gfp^ compared to WT mice (Fig 4B), as the more pronounced disruption of splenic microarchitecture might restrict survival niches for CD8 T cells.

## Discussion

This study demonstrates that activated CD8 T cells are susceptible to NK cell mediated elimination via NCR1 during acute and chronic LCMV infection. Previous reports using LCMV showed that NK cell depletion was associated with the elimination of CD4 T cells, which in turn affected CD8 T cell responses [19]. Others showed that NK cell depletion directly affected CD8 T cells upon LCMV infection [21, 22]. Moreover, IFNAR deficient CD8 and CD4 T cells were recognized and eliminated by NK cells [25] in an NCR1-dependent manner [26]. Depending on the experimental model, different mechanisms were described of how NK cells control T cell responses during virus infections. In MCMV infection, NK cells were reported to specifically kill CD4 T cells in the salivary gland, promoting viral persistence in a TRAIL dependent manner [38]. In contrast, in LCMV infection, NK cells were found to directly eliminate CD8 T cells via the activating receptor NKG2D [21, 22], while others did not find a role for this receptor [19, 26]. The interaction between the inhibitory ligand Qa-1b on T cells / B cells and the NK cell receptor NKG2A led to NK cell inhibition and increased T cell immunity, as NK cell depletion rescued T cell responses in Qa-1b deficient mice [39, 40]. Thus, an immunomodulatory function for NK cells was reported in a number of studies, with NK cells being rheostats for T cell responses [41], but the underlying mechanisms have only partially been resolved. Here, we demonstrate that NCR1 is one major driver in T cell regulation by NK cells during acute and chronic LCMV infection.

In line with the study by Sheppard et al., we observed a slightly increased frequency of mature NK cells (CD11b^hi^ CD27^lo^) in NCR1^gfp/gfp^ mice upon LCMV infection, suggesting a faster maturation process [27]. However, total numbers of CD11b^hi^ CD27^lo^ NK cells in the spleen were comparable between NCR1^gfp/gfp^ and C57BL/6 mice. Further analysis of NK cells in WT and NCR1^gfp/gfp^ mice revealed no alterations in quantity and quality upon LCMV infection (Fig 1). In addition, we obtained the same results in αNCR1 ab treated C57BL/6 mice, excluding a different education of NK cells in absence of NCR1 or that the observed T cell effects are due to impaired effector functions of NK cells in NCR1-deficient mice (S2 Fig, Fig 4).

We found increased LCMV-specific CD8 T cell immunity in NCR1^gfp/gfp^ mice, resulting in more cytokine secreting CD8 T cells and faster control of the virus upon acute LCMV infection (Fig 2). NCR1-mediated regulation of LCMV-specific T cell responses was previously described by Crouse et al., reporting very strong recognition and elimination of IFNAR-deficient LCMV-specific T cells by NK cells in an NCR1-dependent manner. Also in this study, WT CD8 T cells were regulated in an NCR1 dependent NK cell manner, albeit much less pronounced compared to the negative regulation of IFNAR^-/-^ CD8 T cells [26]. The less pronounced regulation of WT CD8 T cells by NK cells in the present study is in agreement with the magnitude of regulation reported in other studies [19, 21]. During acute LCMV infection, activated CD8 T cells (CD44^+^ CD62L^-^) were more abundant in NCR1^gfp/gfp^ compared to WT mice, explaining the increase of total numbers of LCMV-specific CD8 T cells despite comparable frequencies in NCR1^gfp/gfp^, αNCR1 ab treated WT and untreated WT mice. In contrast, during chronic infection, the frequency of virus-specific CD4 and CD8 T cells was highly increased in NCR1^gfp/gfp^ mice, but numbers of virus-specific CD8 T cells and transferred P14 T cells were comparable to WT mice due to a general decrease of total CD8 T cell numbers in NCR1^gfp/gfp^ mice(Fig 4, S6 Fig). In contrast to LCMV-specific CD8 T cells, LCMV-specific CD4 T cell numbers were increased in NCR1^gfp/gfp^ compared to WT mice, and it remains to be addressed why CD4 T cells were numerically less compromised than CD8 T cells during chronic LCMV infection of NCR1^gfp/gfp^ mice.

Moreover, LCMV-specific CD8 T cells were more functional with respect to IFNγ production and degranulation (CD107a) in chronically infected NCR1^gfp/gfp^ mice compared to WT mice. Notably, this enhanced CD8 T cell response in absence of NCR1 resulted in lower viral titers early during chronic infection, faster viral clearance and more severe weight loss in NCR1 deficient mice (Fig 4). Hence, CD8 T cell mediated pathology was increased in absence of NCR1.

Even though NK cells were shown to kill APCs and thereby limit T cell responses in LCMV and MCMV infection [20, 42], we did neither find differences in DC numbers nor expression of co-stimulatory molecules during LCMV infection (S4 Fig). This is in line with previous studies [19, 26], where NK cell depletion did not alter DC numbers or function upon LCMV infection.

*In vivo* cytotoxicity assays, based on transfer of activated polyclonal as well as virus-specific CD8 T cells into LCMV infected recipients with WT or NCR1 deficient NK cells revealed that activated CD8 T cells were eliminated in an NCR1-dependent manner, demonstrating a direct NCR1-mediated regulation by NK cells (Fig 3).

In acute LCMV infection, the absence of NCR1 led to increased LCMV-specific CD8 T cell numbers, which was not the case in a chronic setting. This discrepancy prompted us to compare the lymphoid structure of the spleen between acutely and chronically infected mice. T cell mediated destruction of the lymphoid structure was described to induce immunosuppression [43–45]. A heavily disrupted microarchitecture in the spleen could potentially explain the reduced CD8 T cell numbers in NCR1^gfp/gfp^ mice upon chronic infection. In acute LCMV infection, the splenic organization showed a clear distinction between T and B cell zones in C57BL/6 mice, but more T cells localized to the B cell zone compared to naïve mice. In LCMV infected NCR1^gfp/gfp^ mice, we found more T cells in the B cell zone compared to infected B6 mice, suggesting a more pronounced destruction of the splenic architecture, likely mediated by cytotoxic T cell activity. However, LCMV-specific P14 T cells were found at comparable numbers in the B cell zones in acutely infected C57BL/6 and NCR1^gfp/gfp^ mice, possibly explaining the observation that NCR1 deficient mice exhibited increased overall CD8 T cell numbers upon acute LCMV infection due to maintenance of a less damaged T cell niche.

In contrast, chronic LCMV infection resulted in severe disruption of the splenic microarchitecture, in particular T cell localization was severely dispersed in NCR1 deficient mice, as more T cells and P14 T cells localized to the B cell zone in NCR1^gfp/gfp^ mice (Fig 5). We suggest that the more efficient CD8 T cell response present in NCR1 deficient mice mediated severe pathology, leading to damage of the secondary lymphoid organ structure and a possibly severely compromised CD8 T cell niche. We therefore propose that NK cells recognize and eliminate highly activated CD8 T cells via NCR1 to protect the host from an overshooting CD8 T cell response. This reinforces the notion that NK cells serve as rheostats regulating the pool of activated CD8 T cells, which might be harmful for the host, and this regulation can be mediated via NCR1. Recent studies found that the absence of NCR1 leads to failure of TRAIL expression [31, 32]. Therefore, the effects observed in studies using NCR1 deficient mice could theoretically be TRAIL dependent [6, 9, 46–50]. To exclude this, we analyzed NCR1 and TRAIL expression in NCR1^gfp/gfp^ and αNCR1 ab treated B6 mice and confirmed absence of TRAIL expression in NK cells from NCR1^gfp/gfp^ mice. However, αNCR1 ab treated mice had similar TRAIL expression as WT mice (S2 Fig). As we obtained similar results using NCR1^gfp/gfp^ or αNCR1 treated B6 mice, we exclude TRAIL-mediated regulation of virus-specific CD8 T cell responses in acute or chronic LCMV infection, in line with a previous report studying LCMV infection in TRAIL deficient mice [51].

Other studies investigated the role of NCR1 expressing NK cells on the outcome of various disease settings. For instance, NCR1 was reported to be essential for the reduction of graft versus host disease [50], although it remains unclear whether NCR1 acts directly on host-reactive T cells or if the regulation is indirect via APCs. In human hepatitis C virus (HCV) infection, decreased levels of NKp46 were associated with persistent infection, which allows speculation whether the host decreases NKp46 expression on NK cells to protect activated virus-specific T cells [52]. Furthermore, tumor infiltrating T cells (TIL) were negatively regulated by NKp46 expressing innate lymphoid cells. The addition of αNKp46 ab to ILCs and TILs resulted in enhanced T cell numbers similar to TILs cultured in absence of ILCs [53]. Thus, our findings that NK cells negatively regulate virus-specific CD8 T cell responses via NCR1 in a direct manner may not only account for virus infections, but might be extended to allo-reactivity or tumor settings. Blocking NCR1 on NK cells might therefore be a suitable tool to boost T cell responses in chronic viral infections and cancer.

## Materials and methods

### Ethics statement

This study was conducted in accordance to the guidelines of the animal experimentation law (SR 455.163; TVV) of the Swiss Federal Government. The protocol was approved by Cantonal Veterinary Office of the canton Zurich, Switzerland (Permit number 147/2014 and 115/2017).

### Mice

The mice were housed and bred in specific pathogen-free facilities. The following strains were used: C57BL/6J mice (WT, B6), purchased from Janvier Elevage (Le Genest Saint Isle, France). P14 transgenic (Ly5.1^+^) mice expressing a TCR specific for LCMV peptide gp_33-41_ described previously [54]. Smarta transgenic (Ly5.1^+^) mice expressing a TCR specific for LCMV peptide gp_61-80_ were described earlier [55]. In NCR1^gfp/gfp^ mice, the endogenous Ncr1 gene is replaced by GFP [6]. All mice were used at age 6 to 14 weeks.

### Viruses, viral peptides and blocking antibodies

The LCMV isolates WE, the mutant strain LCMVWE 8.7 (LCMV8.7) [54], LCMV docile and LCMV clone 13 were provided by Dr. R.M. Zinkernagel (University Hospital, Zurich, Switzerland). The viruses were propagated at low multiplicity of infection on L929 cells (LCMV WE), LCMV-docile was propagated on MDCK cells and LCMV clone 13 strains were propagated on BHK-21 cells. Recombinant vaccinia virus expressing the LCMV glycoprotein gp33 (VVG2) was provided by Dr. D.H.L. Bishop (Oxford University, Oxford, U.K.) and was grown on BSC40 cells at low multiplicity of infection. Co-infection of LCMV WE 8.7 and VVG2 was performed by i.v. injection of 1x10^4^ffu LCMV WE 8.7 and 5x10^6^pfu VVG2. LCMV WE infection was performed using 200 ffu and injections with LCMV docile and LCMV clone 13 were performed with 1.5x10^6^ ffu and 2x10^6^ ffu, respectively i.v. The viral peptide gp_33-41_ (KAVYNFATM) and np_396-404_ (FQPQNGQFI) were purchased from NeoMPS (Strasbourg, France). Blocking NCR1 was performed by i.p. injections of 200μg αNCR1 (NCR1.15mAb, IgG1 κ) (generously provided by Prof. Stipan Jonjic, University of Rijeka, Croatia) one day before and after infection, and afterwards twice a week. Isotype control was applied using 200μg of Ultra-LEAFTM Purified Mouse IgG1 κ (Biolegend, UK) one day before and after infection, and afterwards twice a week.

### Adoptive transfer

CD8^+^ T cells were isolated from naïve P14 mice using positive selection with CD8^+^ beads (Miltenyi Biotech, Bergisch Gladbach, Germany) according to manufacturer’s protocol and adoptively transferred into recipient mice 1 day prior to virus infection. CD4^+^ T cell were isolated from naïve Smarta mice using positive selection with CD4^+^ beads (Miltenyi Biotech, Bergisch Gladbach, Germany) according to manufacturer’s protocol and adoptively transferred into recipient mice 1 day prior to virus infection.

### Flow cytometry and lymphocyte stimulation

Lymphocytes were harvested from spleen, lung and lymph nodes as previously described [56]. For DCs, enzymatic digestion of the spleen using Liberase and DNAse (Roche Diagnostics, Basel, Switzerland) was performed for 15 min at 37°C. Blood samples were taken from the tail vein and were treated with ACK lysis buffer for 5 min at RT in order to lyse erythrocytes. Surface stainings were performed in PBS for 20 min at RT. APC and PE-conjugated MHC class I tetramers were generated as previously described [57]. Tetramer staining was performed in PBS and incubated for 30 min RT. To measure cytokines in T cells, splenocytes were resuspended in 10% FCS containing RPMI and 1 μg/ml of peptide and 10 μg/ml Brefeldin A (Lucerna Chem AG) to re-stimulate the cells in vitro for 6 h at 37°C followed by a washing step and surface staining. CD107a-FITC (1D4B) (BioLegend) was directly given to the culture due to its high turnover rate. The cells were fixed using Fixation/Permeabilization Buffer consisting of 2xBD lysing solution (BD Biosciences) and 0.08% Tween-20 (Chemie Brunschwig AG). Intracellular cytokine stainings were incubated for 30 min at RT and samples were washed afterwards. Intracellular NK cell staining was done as described before [58]. Antibodies used for stainings are the following and were purchased from BioLegend (CD3-PE (145-2-C11), CD45.1-APC (A20), CD45.1-PE (A20), TNF-FITC (MP6-XT22), CD8-PerCP (53–6.7), CD11b-PerCP (M1/70), CD27-PE (LG.3A10), NK1.1-PE (PK136), NK1.1-APC-Cy7 (PX136), NK1.1-BV711(PK136), panNK-APC (DX5), CD44-BV510 (IM7), I-A/I-E-PerCP(M5/114.15.2), CD11c-BV421 (N418), CD86-APC (GL-1), CD40-PE-Cy7 (323), CD80-FITC (16-10A)), from BD-biosciences (CD3-BV510 (145-2C11), IFNγ–C(XMG1.2)), eBioscience (IFNγ–PE-Cy7 (XMG1.2)), Bioss antibodies (Properdin-Alexa Fluor647 (polyclonal) and Molecular Probes (Granzyme B (PE-MP6-XT22)). Viability of cells were determined by using fixable near-IR dead cell staining (Life Technologies). Data was acquired on a LSRII™, Canto™ and Fortessa™ flow cytometer (BD Bioscience, Switzerland) and analyzed with FlowJo software (Treestar, Ashland, OR, USA).

### Determination of virus titers

Determination of virus-titers was done as described before [59].

### YAC-1 in vitro killer assay

Activated NK cells were harvested from donor mice 2 days after LCMV WE infection and isolated using DX5^+^ magnetic beads (Miltenyi Biotech,Bergisch Gladbach, Germany). 20’000 YAC-1 cells were co-cultured with DX5^+^ NK cells in triplicates in a ratio of 1:1, 1:10 and 1:25. After 4 h incubation at 37°C, surface stainings were performed in PBS and the samples were washed. The apoptotic markers, Annexin V-PB (BioLegend UK Ltd) and 7AAD (BD Biosciences) were diluted in Annexin V Buffer (BD Biosciences) and incubated for 30 min at 4°C followed by transfer on ice and direct acquisition of the samples.

### In vivo killer assay for polyclonal CD8 T cells

Activated CD8 T cell were obtained from day 7 LCMV WE infected NCR1^gfp/gfp^ mice and CD8 T cells were positively MACS sorted. To distinguish the donor CD8 T cells from the host, donor CD8 T cells were CTV (CPDeF450) (Thermo Fisher, Waltham, USA) labeled before transfer. For this, CD8 T cells were washed in PBS and resuspended in 10 μM CTV and incubated for 10 min at 37°C followed by addition of FCS to quench the labeling. Cells were washed twice in PBS containing 5% of FCS. 8x10^6^ labeled CTV^+^ CD8^+^ T cells were adoptively transferred into day 2 1x10^6^ ffu LCMV WE infected recipient mice. After 4 h, lymphocytes of spleen and lung were isolated and analyzed by flow cytometry.

### In vivo killer assay of virus-specific CD8 T cells

Activated virus-specific CD8 T cells (P14 T cells) were obtained by transfer of 1x10^6^ naïve P14 T cells into NCR1^gfp/gfp^ mice following co-infection with LCMV8.7 and VVG2. After 5 days, splenic CD8 T cells were positively MACS sorted and transferred into day 3 LCMV WE infected recipient hosts, following a 4 h incubation. Spleens were harvested and the recovered number of P14 T cells was assessed. Prior to transfer, the amount of P14 T cells among all CD8 T cells from donor mice was analyzed by flow cytometry.

### Immunohistochemistry and fluorescence microscopy

10 μm thin sections were prepared from frozen spleens embedded in Optimal Cutting Temperature (O.C.T.) compound (Sakura). The sections were air-dried and fixed in ice-cold acetone for 10 min at RT followed by a washing step with PBS. The samples were blocked using 10% FCS in PBS for 1 h at RT. After washing with PBS, antibody dilutions were performed in PBS containing 1% FCS (B220-APC, CD3-PE (145-2-C11) and CD45.1-PB(A20) (BioLegend)). After incubation for 1 h in the dark, the sections were washed with PBS and the slides were mounted with Mowiol (Sigma-Alrich Chemie GmbH). The next day, images were acquired on the Visitron Confocal System inverse confocal microscope, Visitron Systems GmbH) with 10x magnification (10x objective, aperture 0.5, Immersion: Air, Contrast: Ph1) at RT. Acquisition of the pictures was performed using Evolve 512 EMCCD cameras (Photometrics). Data was analyzed using Volocity software (Version 6.3, PerkinElmer).

### Statistical analysis

Statistical significance was determined by two-tailed unpaired *t* test, unpaired Mann-Whitney U-test and multiple time points were analyzed applying two-way ANOVA followed by Sidak’s multiple comparisons test using GraphPad (La Jolla, CA, USA). Statistical significance was determined with *p< 0.05, **p< 0.01, ***p< 0.001 and ****p< 0.0001.

## Supporting information

S1 Fig Increased number of virus-specific CD8 T cells in αNCR1 blocking ab treated C57BL/6 mice. (TIF)

S2 Fig Blocking of NCR1 does not affect TRAIL expression on NK cells. (TIF)

S3 Fig Absence of properdin on virus-specific CD8 T cells. (TIF)

S4 Fig Number and expression of co-stimulatory molecules on DCs in NCR1^gfp/gfp^ and WT mice. (TIF)

S5 Fig Increased frequency and numbers of virus-specific CD4 T cells in chronic LCMV infection in absence of NCR1. (TIF)

S6 Fig Heterozygous NCR1^gfp/+^ mice do not show an increased CD8 T cell response. (TIF)
