# Supplementary figures and images for: NK cells negatively regulate CD8 T cells via natural cytotoxicity receptor (NCR) 1 during LCMV infection
Source: PLoS Pathog. 2019 Apr 17;15(4):e1007725. doi: 10.1371/journal.ppat.1007725 (PMC6469806; doi:10.1371/journal.ppat.1007725)

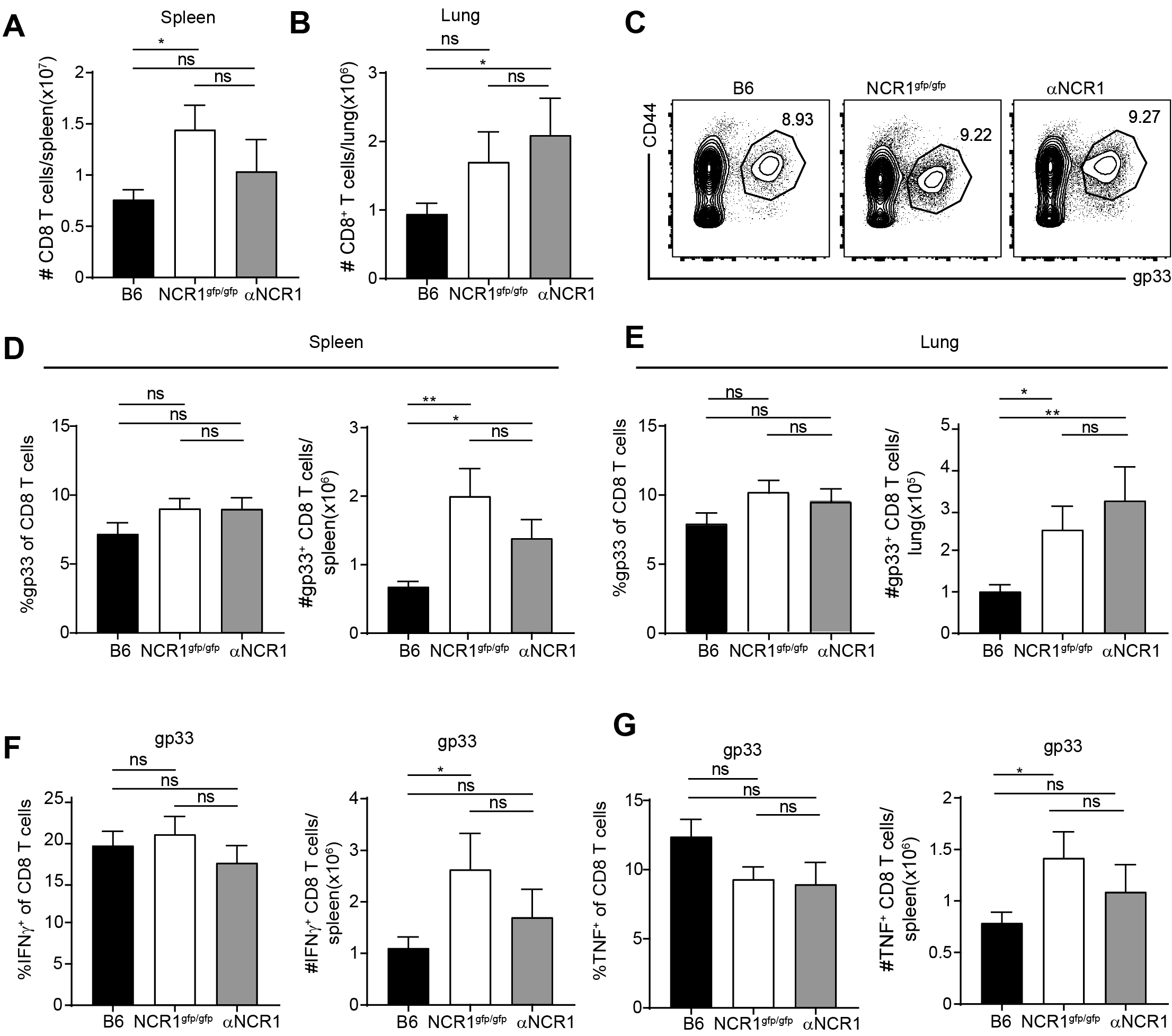

Supplement: S1 Fig — (TIF) [file ppat.1007725.s001.tif]

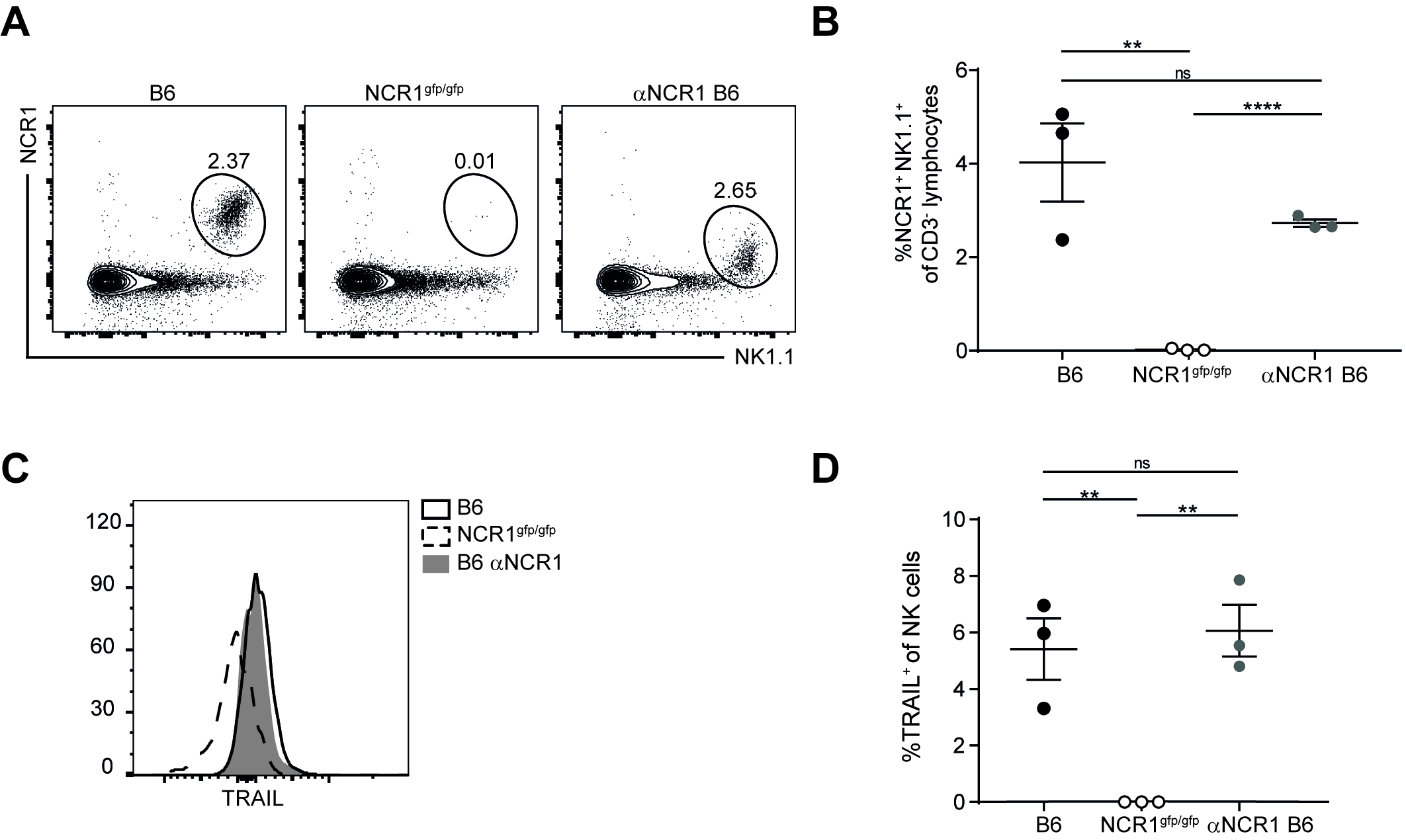

Supplement: S2 Fig — (TIF) [file ppat.1007725.s002.tif]

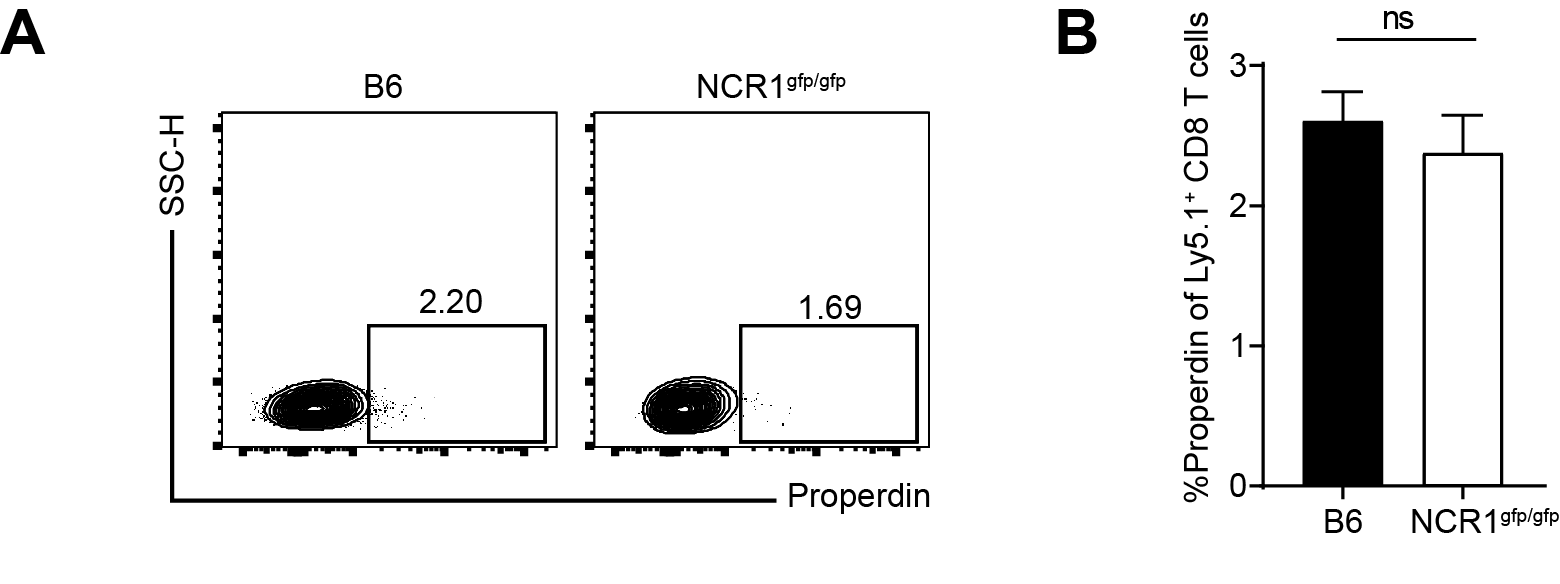

Supplement: S3 Fig — (TIF) [file ppat.1007725.s003.tif]

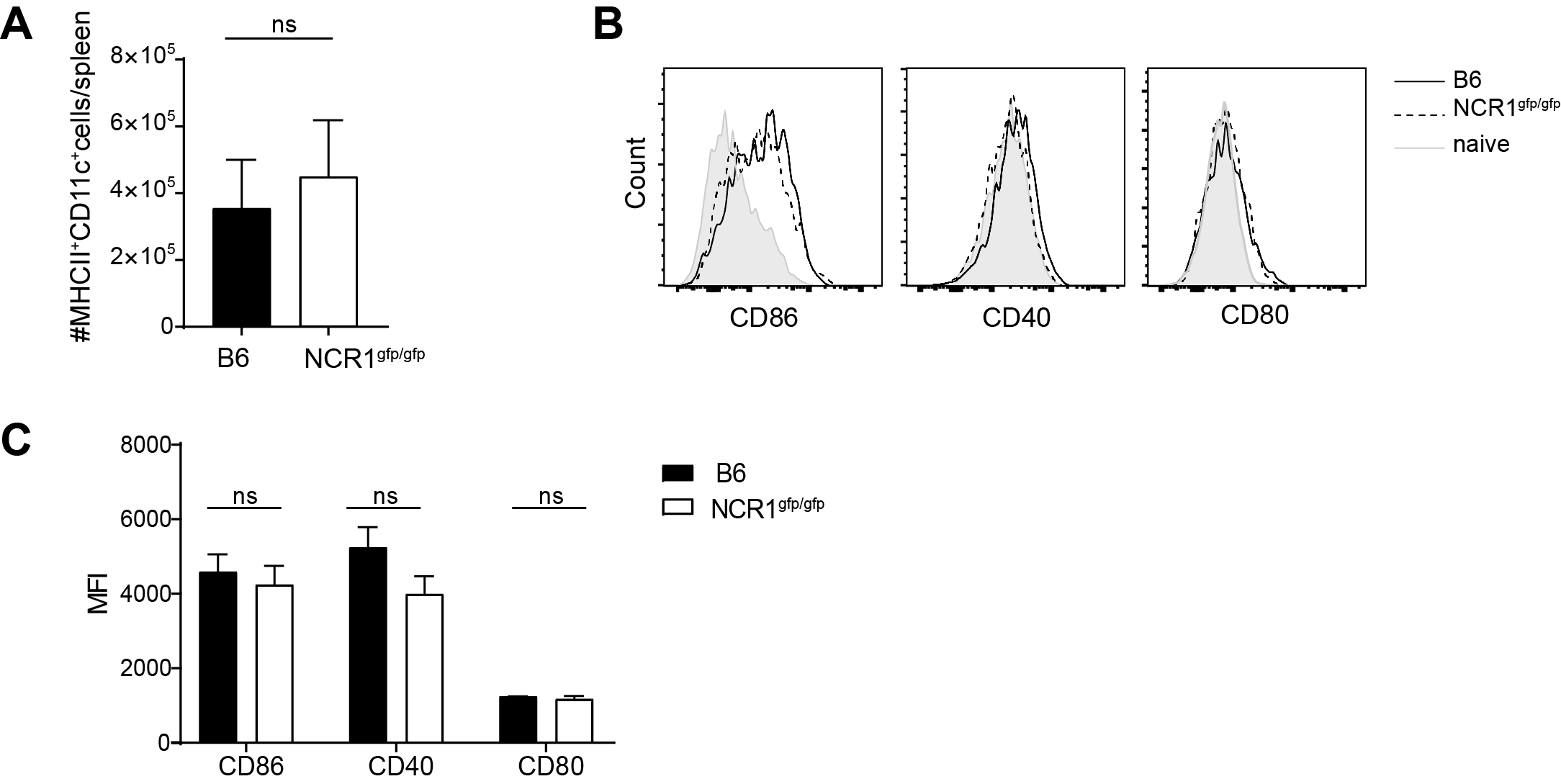

Supplement: S4 Fig — (TIF) [file ppat.1007725.s004.tif]

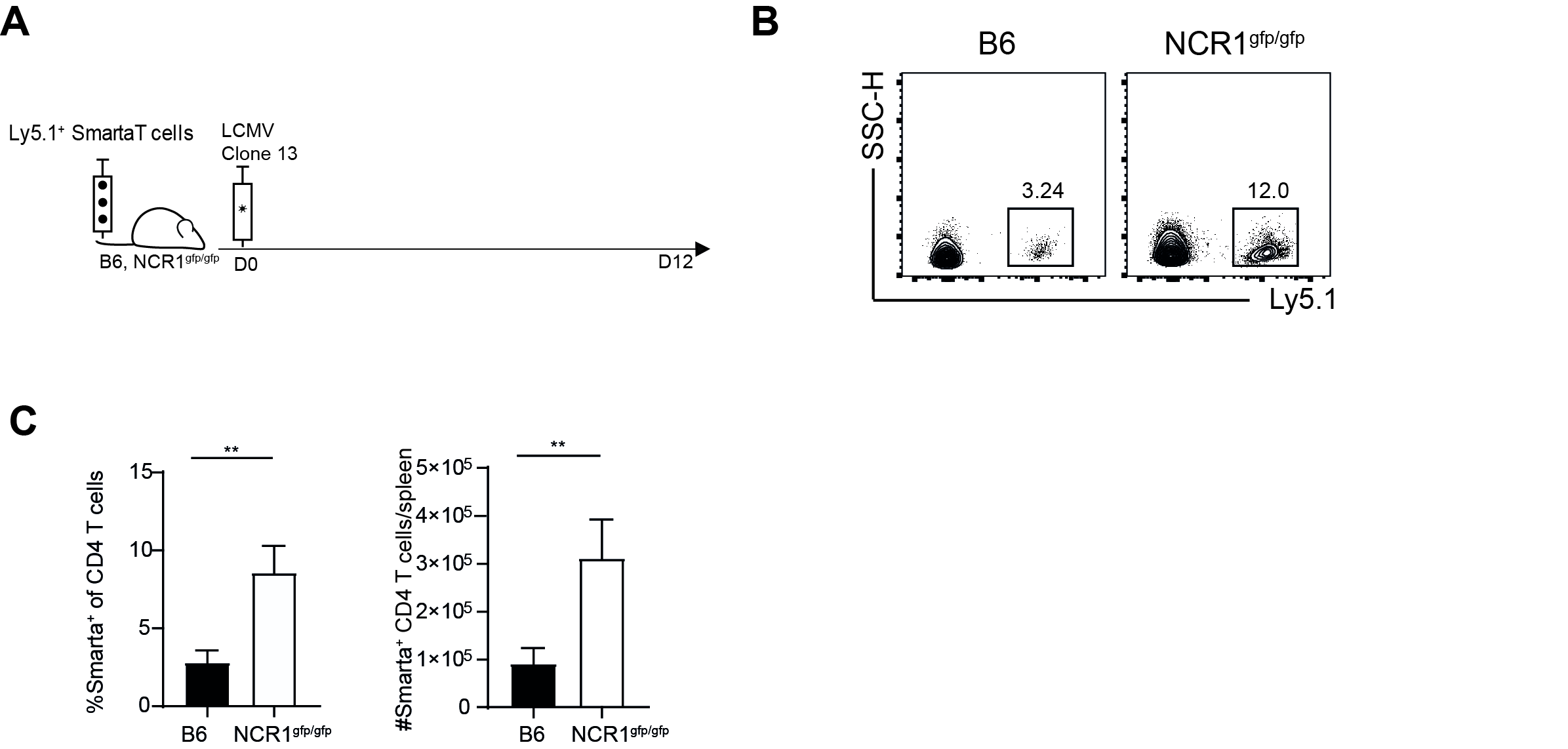

Supplement: S5 Fig — (TIF) [file ppat.1007725.s005.tif]

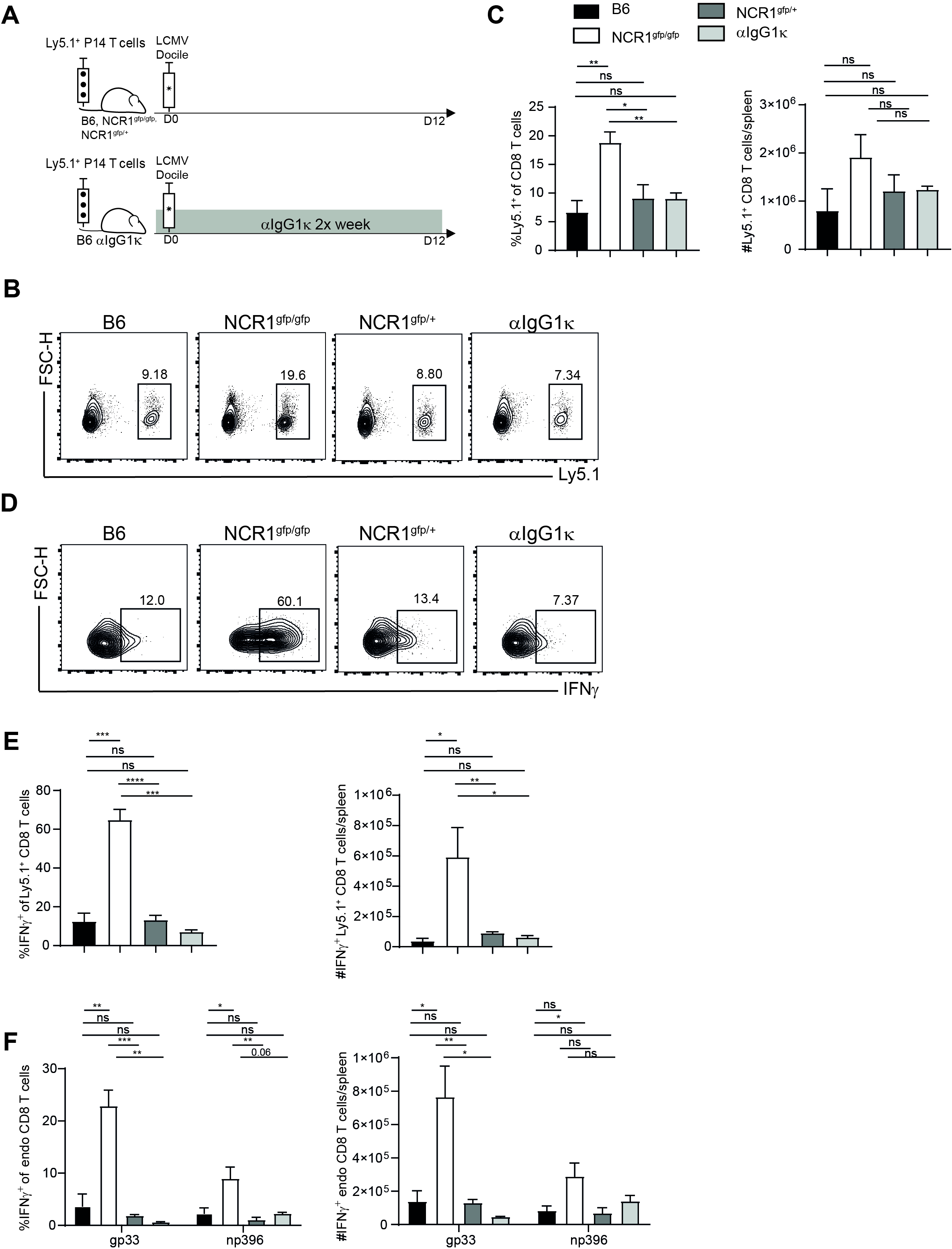

Supplement: S6 Fig — (TIF) [file ppat.1007725.s006.tif]
